# Impact of air pollution and climate change on maternal, fetal and postnatal health

**DOI:** 10.1016/j.jped.2024.10.006

**Published:** 2024-11-22

**Authors:** Mariana Matera Veras, Paulo Hilário Nascimento Saldiva

**Affiliations:** Hospital das Clínicas da Faculdade de Medicina da Universidade de São Paulo (HCFMUSP), Departamento de Patologia, Laboratório de Patologia Ambiental e Experimental – LIM05, São Paulo, SP, Brazil

**Keywords:** Particulate matter, Temperature, Extreme wheatear, Pregnancy, Fetal development

## Abstract

**Objective:**

Besides socioeconomic factors, environmental pollution, and climate change are contemporary threats to health. In this review, the authors present results from a recent comprehensive synthesis of existing research on the effects of air pollution and climate change on gestation, fetal development, and postnatal health.

**Data sources:**

Findings from systematic reviews conducted over the past five years and available in PubMed were used.

**Summary of findings:**

A vast and robust evidence exists on the association between air pollution exposures and negative outcomes. Gestational diabetes, hypertensive disorders of pregnancy, preeclampsia, spontaneous abortion, and maternal postpartum depression are reported. Fetal development and postnatal health are also impaired by exposures; low birth weight is a common finding from studies worldwide, but there are increased risks for malformations and impairments in neurodevelopment. While there are fewer studies on factors related to climate change, there is sufficient evidence regarding the direct and indirect effects on maternal health and fetal development. Increased risks for prematurity, low birth, and emergency room visits are associated with higher temperatures. Asthma incidence and infectious respiratory disease risks are also influenced by extreme weather events. It is essential to recognize the profound impact that environmental factors, such as air pollution and climate change can have on maternal health, fetal development, and neonatal health.

**Conclusion:**

The data presented underscores the significant risks that environmental pollution poses during gestation, influencing not only maternal health but also the short- and long-term well-being of the child.

## Introduction

Understanding the interplay between environmental factors and gestation is essential for developing effective public health strategies aimed at improving maternal and fetal health outcomes. Addressing issues like access to healthy foods, socioeconomic disparities, and stress management can significantly mitigate risks associated with adverse pregnancy outcomes.^1^ Besides socioeconomic factors, environmental pollution and climate change are contemporary threats to a healthy gestation and full development of the fetus and the health of the neonate.^2^^,^^3^ In this review the authors present results from a recent comprehensive synthesis of existing research on the effects of air pollution and climate change on gestation, fetal development and postnatal health.

## Methods

In this review the authors included the most recent data from systematic reviews retrieved from PubMed published in the last 5 years, using the search terms combination: *air pollution AND gestation OR pregnancy* and *climate change AND gestation OR pregnancy*. The authors began by screening titles and abstracts to determine their eligibility for inclusion. Studies on the effects of wildfire smoke were excluded. Following this, the authors conducted a thorough review of the full texts of the articles that were not excluded. The selected articles were grouped according to the outcome evaluated. Based on this classification, three topics were defined to build this review: (I) Adverse effects on maternal health and gestation, (II) Impairments of fetal development, and (III) Negative impacts on postnatal health. A brief introduction on climate change and air pollution is included.

## Air pollution and climate change

Air pollution and climate change are interconnected issues that stem from similar sources and have compounding effects on both the environment and public health.^4^ The burning of fossil fuels for energy, transportation, and industrial processes is the primary source of both air pollution and greenhouse gas emissions. This results in the release of harmful pollutants such as carbon dioxide (CO_2_), methane (CH_4_), particulate matter (PM), nitrogen dioxide (NO_2_), sulfur dioxide (SO_2_), carbon monoxide (CO) and ground-level ozone (O_3_), which contribute to climate change while simultaneously degrading air quality.^4^ Indoor air pollution should not be forgotten, it affects around 2.4 billion people who rely on solid fuels for cooking and heating.^5^

Both outdoor and indoor air pollution, pose a significant threat to public health, particularly for vulnerable populations such as children under five years old. According to the World Health Organization (WHO), approximately 99% of the global population breathes air that exceeds recommended safety limits, with low- and middle-income countries experiencing the highest levels of exposure.^3^

Additionally, the authors are witnessing an increasing number of wildfires, which pose significant threats to human health, particularly for vulnerable populations such as children, unborn infants, and the elderly. According to UNICEF, nearly 677,745 premature deaths globally are attributed to smoke from landscape fires each year, with around 40% of these fatalities occurring in children under five years old.^6^ However, it is not in the scope of this review the effects of gestational exposure to wildfire smoke, a brief overview on this topic was recently published.^7^

Climate change is also a critical public health issue, particularly concerning its effects on pregnancy and child health. As global temperatures rise and extreme weather, events become more frequent, pregnant women and their developing fetuses face heightened risks associated with environmental changes.

## Results

The authors retrieved 149 manuscripts and after removal of duplication and those out of the scope of this review, we ended with 86 studies. Due to limitations on the number of references, the most recent reviews were included. The list of all studies retrieved and consulted for this review can be accessed via this link: https://www.ncbi.nlm.nih.gov/sites/myncbi/1ravwbM1gdKkRJ/collections/64501585/p and in Table 1 the authors have summarized the key findings from the studies cited in this review.

**Table 1:** Summary of key findings from selected Studies on the effects of air pollution and climate change on maternal, fetal and neonatal health.

Author	Purpose	Study design	Outcome
Mazumder H, Rimu FH, Shimul MH, et al. (2024)	Summarize the global evidence on the effects of air pollutants on maternal health outcomes.	umbrella review	Ambient air pollutants are positively associated with Gestational Diabetes Mellitus (GDM), Hypertensive Disorders of Pregnancy (HDP) and Preeclampsia (PE). Limited evidence of positive correlation with Gestational Hypertension (GH) and Spontaneous Abortion (SAB) and Postpartum Depression (PPD).
Liang Y, Li M, Lyu Q, et al. (2024)	Investigate the relationship between long-term and short-term maternal exposure to air pollution and PROM.	systematic review and meta-analysis	Long-term maternal exposure in the second or third trimester and short-term maternal exposure to ambient air pollution can increase the risk of PROM.
Pappas A, Kovats S, Ranganathan M. (2024)	Examine the current literature on the impact of extreme weather events (EWE) on maternal health.	scoping review	EWE negatively impact maternal health through various mechanisms including access to services, stress and mortality. Little evidence is available for low-income countries.
Veenema RJ, Hoepner LA, Geer LA. (2023)	Identify research on the effects of climate change-related environmental exposures on perinatal and maternal health in the United States.	systematic literature review	High and low-temperature extremes negatively influence neonate and maternal health. Adverse pregnancy outcomes were linked to hurricanes, tropical cyclones, and flash floods.
Parasin N, Amnuaylojaroen T, Saokaew S. (2024)	Investigate the impact of PM2.5 exposure during pregnancy on LBW.	systematic review and meta-analysis	The findings revealed a significant relationship between PM2.5 exposure and LBW in both the first and second trimesters.
Huang Z, Wu J, Qiu Y, et al. (2023)	Explore the correlation between ambient air pollution and the occurrence of neonatal orofacial deformity (OFC).	systematic review and meta-analysis	There is a significant statistical correlation between exposure to PM10, PM2.5, O_3_ and the risk of OFCs in the second month of pregnancy.
Gholami Shahrebabak M, Mobasheri-Shiri M, Hesari M, et al. (2024)	Investigate the association between maternal exposure to air pollutants and outcomes of in vitro fertilization (IVF).	systematic review and meta-analysis	Exposure to air pollutants was linked to the risk of ectopic pregnancy and has a reverse association with biochemical pregnancy. There is also a negative association between exposure live birth rates.
Chersich MF, Pham MD, Areal A, et al. (2020)	Assess whether exposure to high temperatures in pregnancy is associated with increased risk for preterm birth, low birth weight, and stillbirth.	systematic review and meta-analysis	Odds of a preterm birth raise 1.05-fold per 1 °C increase in temperature and 1.16-fold during heatwaves. Higher temperature are associated with reduced birth weight. Stillbirths increase 1.05-fold (per 1 °C rise in temperature. Associations were larger among women in lower socioeconomic groups and at age extremes.
Daba C, Asmare L, Demeke Bayou F, et al. (2024)	Determine the association between indoor air pollution exposure and adverse pregnancy outcomes in low and middle-income countries.	systematic review and meta-analysis	Exposure to indoor air pollution increased the risk of small for gestational age by 23.7% followed by low birth weight (17.7%).
Brink N, Lakhoo DP, Solarin I, et al.(2024)	Assess evidence on the long-term impacts on the fetus of heat exposure in utero.	systematic review	Heat exposure during pregnancy is associated with decreased earnings and lower educational attainment, worsened cardiovascular, psychiatric and anthropometric later in life. The effect on female was greater than on males.
Weeda LJZ, Bradshaw CJA, Judge MA, et al. (2024)	Analyze various child health outcomes due to climate change.	systematic review and meta-analysis	Exposure to extreme temperature increases the risk (60 % on average) of preterm birth Respiratory disease, mortality, and morbidity, are also influenced by climate changes.
Luben TJ, Wilkie AA, Krajewski AK, et al. (2023)	review the available literature for epidemiologic evidence of the association between short-term air pollution exposure and infant mortality.	systematic review and meta-analysis	Increased exposure to air pollutants is associated with infant mortality.
Feng Y, Liu X, Zhang X, et al. (2024)	Evaluate recent epidemiological evidence on the association of air pollution with congenital anomalies (CA).	systematic review and meta-analysis	10 μg/m3 increase in PM10 exposure during the 1st month of pregnancy and at the first trimester is associated with increased overall CAs. Exposure during the whole gestation is associated with congenital heart disease, patent ductus, chromosomal anomalies and nervous system anomalies.
Amnuaylojaroen T, Parasin N, Saokaew S. (2024)	Investigate the association between air pollution and the vulnerability of children to autism spectrum disorders (ASD).	systematic review and meta-analysis	The findings demonstrated a correlation between exposure to PM10 and PM2.5 and the occurrence of ASD.
Zhao J, He T, Wang F, et al. (2024)	Investigate the relationship between both prenatal and postnatal exposures to particular air pollutants and clinically diagnosed ADHD (attention deficit hyperactivity disorder).	systematic review	Results indicate a strong connection between prenatal exposure to PM2.5 and NOx and a heightened risk of ADHD,
Yue D, Shen T, Mao J,et al. (2022)	Investigate the association between air pollution exposure during pregnancy and the risk of eczema in offspring.	systematic review and meta-analysis	A significant association between the maternal exposure to NO_2_ and childhood eczema was observed, but no association was observed between exposure to PM10, PM2.5, and SO_2_ and the risk of eczema in offspring.
Liu Y, Li Y, Xu H, et al. (2023)	Investigate the association of pre- and postnatal PM exposure with blood pressure (BP) and hypertension in children and adolescents.	systematic review and meta-analysis	Prenatal and postnatal exposure to PM can increase BP, and contribute to an increased prevalence of hypertension in children and adolescents.
Margetaki K, Bempi V, Michalaki E, et al. (2024)	Investigate the association of prenatal exposure to air pollution with offspring obesity and evaluate the possible protective effect of maternal fruits and vegetables consumption.	epidemiological	Maternal consumption of fruits and vegetables (protective factor) influence the association between air-pollution and childhood obesity.
Siewert B, Kozajda A, Jaskulak M, et al. (2024)	Investigate relationship between various air pollutants and childhood obesity.	systematic review	In places with low air pollution, influence of exposures is low or not existent. In places with high levels, there is a relationship for PM2.5, PM1, PM10, NO_x_, and SO_2_ with childhood obesity.
Herting MM, Younan D, Campbell CE, Chen JC.(2019)	Evaluate and synthesize the reported evidence from MRI studies on how early-life exposure to outdoor air pollution affects neurodevelopment.	systematic review	Associations between exposure and smaller white matter surface area, microstructure, region-specific patterns of cortical thinness) and smaller volumes and/or less density within the caudate (n = 3); altered resting-state functional connectivity and brain activity to sensory stimuli (n = 1).
Castagna A, Mascheroni E, Fustinoni S, et al. (2022)	Evaluate the association between air pollutant exposure in prenatal and/or postnatal periods and specific neurodevelopmental skills in preschool- and school-age children.	systematic review	Negative impacts of air pollutants on children's neurodevelopmental abilities were noted, though these effects did not result in clinically significant performance deficits. The areas most affected included overall intellectual functioning and attention/executive functions. Share.
Mishra S, Stukken CV, Drury S, et al. (2024)	Investigate using population-based studies the association between prenatal air pollution exposure and Telomere length (TL) and mitochondrial DNA (mtDNA) content at birth.	systematic review	Results show evidence that prenatal PM2.5 exposure affects the telomere-mitochondrial axis of aging at birth.
Martens DS, Cox B, Janssen BG, et al. (2022)	Assess the association of prenatal exposure to particulate matter (PM) with newborn telomere length.	cohort	Cord blood and placental telomere length were significantly and inversely associated with PM2.5 exposure during midgestation.
Dalugoda Y, Kuppa J, Phung H, et al. (2022)	Provide an overview of the current evidence of the association between elevated ambient temperature exposure during pregnancy and adverse maternal, fetal, and neonatal outcomes.	scoping review	There is a heightened risk associated with higher temperature exposures. However, there has been less focus on the connections between heat and various other negative outcomes, including congenital anomalies and neonatal mortality.

### Adverse effects on maternal health and gestation

Maternal exposure to air pollution during pregnancy has been linked to negative maternal health outcomes. Mazumder et al.^[^^8^^]^ conducted an umbrella review to systematically summarize the global evidence on the effects of air pollutants on maternal health. They found that PM, SO_2,_ and NO_2_ have positive associations with gestational diabetes mellitus (GDM). Additionally, PM and NO_2_ consistently showed a positive relationship with hypertensive disorders of pregnancy (HDP) and preeclampsia (PE). Although the evidence is limited, there is a noted correlation between PM and gestational hypertension (GH) as well as spontaneous abortion (SAB). Only one meta-analysis examined the impact of air pollution on maternal postpartum depression (PPD), finding a significant positive relationship with PM10 (inhalable particles, with diameters that are generally 10 micrometers)

Exposure to air pollution may also predispose to premature rupture of membranes (PROM). Maternal exposure to PM2.5 (inhalable particles, with diameters that are generally 2,5 micrometers or less) during the second trimester is associated with an increased risk of preterm premature rupture of membranes (PROM), with a pooled odds ratio of 1.15 (95% CI: 1.05–1.26). Additionally, maternal exposure to PM10, NO2, NO, carbon monoxide CO, and sulfur dioxide (SO_2_) throughout pregnancy, as well as short-term exposure to PM2.5, NO2, SO2, and ozone (O_3_), is also linked to the occurrence of PROM.^9^

The mechanisms by which air pollution affects blood pressure during pregnancy it is not clear, but may involve oxidative stress and inflammation, leading to endothelial dysfunction and impairments in placental function.^10^

Pregnant women are particularly vulnerable to the impacts of climate change due to physiological changes that occur during this period. These changes can impair their ability to cope with environmental stressors, making them more susceptible to heat stress and related complications. Moreover, climate change can indirectly affect maternal and child health by disrupting food security and access to healthcare. Extreme weather events can compromise food production and distribution, leading to nutritional deficiencies that may adversely impact fetal development. Additionally, the psychological effects of climate-related disasters can contribute to mental health issues in pregnant women, further complicating their health status.^11^

There are few studies on the effects of climate change-associated events and maternal health. A recent systematic review^12^ based on studies from the United States examined the effects of extreme heat, air pollution, and natural disasters on maternal health. Results indicate that low and high-temperature extremes negatively influence perinatal and maternal health (reduced gestational age, increased risk of early term birth, or increased risk of preterm birth). Reviewed studies indicate that reduced length of gestation, preterm birth, depression, hypertension and gestational diabetes mellitus, and increased emergency department visits for pregnancy complications were also associated with hurricanes, tropical cyclones, and flash flood events. Furthermore, extreme events can impair access to prenatal care and availability and usage of maternity care and support services, ultimately leading to negative perinatal outcomes and threats to maternal health.

### Impairments of fetal development

One of the most frequent negative outcomes associated with gestational exposure to air pollution is LBW. Studies from different parts of the world confirm this association, although studies do not always agree on which trimester the exposure could be more detrimental. One of the most recently published Meta-analysis^13^ indicated a significant association between PM2.5 exposure and low birth weight (LBW) during both the first and second trimesters (OR 1.05, 95% CI 1.00–1.09, *p* < 0.001). No significant difference was observed between the trimesters (*p* = 0.704). These results highlight the ongoing impact of PM2.5 on fetal development throughout all stages of pregnancy.

The risk of congenital malformations such as orofacial clefts in newborns is also heightened due to exposure to specific air pollutants during pregnancy, raising concerns about the potential for serious developmental issues.^14^

Couples seeking assisted reproductive technology (ART) also face the adverse effects of air pollution exposure. According to Gholami et al.^15^ This exposure is linked to an increased risk of ectopic pregnancies and a decline in live birth rates.

Environmental temperature is also an important factor for a successful gestation, Cherisich et al.^16^ reviewed 70 studies from 27 countries and they found that odds of a preterm birth rose 1.05-fold (95% confidence interval 1.03 to 1.07) per 1 °C increase in temperature and 1.16-fold (1.10 to 1.23) during heatwaves. Higher temperature was associated with reduced birth weight in 18 of 28 studies, with considerable statistical heterogeneity. Eight studies on stillbirths all showed associations between temperature and stillbirth, with stillbirths increasing 1.05-fold (1.01 to 1.08) per 1 °C rise in temperature. Associations between temperature and outcomes were largest among women in lower socioeconomic groups and at age extremes. However, they point out that differences in methodology to assess exposure limit the comparison between studies.

Indoor air pollution (IAP) is a significant concern in low- and middle-income countries (LMICs), particularly regarding its impact on gestational outcomes. In these regions, many households rely on solid fuels for cooking and heating, leading to high levels of indoor pollutants such as particulate matter (PM2.5), CO, and volatile organic compounds. Daba e cols.^17^ found that the pooled association between IAP and at least one adverse pregnancy outcome was 15.5% (95%CI: 12.6–18.5). Exposures increased the risk of small for gestational age by 23.7% (95%CI: 8.2–39.3) and low birth weight by (17.7%; 95%CI: 12.9–22.5). The implications of indoor air pollution are especially critical LMICs, where healthcare resources may be limited.

### Negative impacts on postnatal health

Climate change, particularly global warming, is amongst the greatest threats to human health. While short-term effects of heat exposure in pregnancy, such as preterm birth, are well documented, long-term effects (health and social) have received less attention. Brink et al.^18^ reviewed 29 studies, all studies were observational, comprising 17 cohorts, 5 case-control, and 8 cross-sectional designs, with data spanning from 1913 to 2019 and participants ranging from neonates to the elderly. They found that increased heat exposure during pregnancy was linked to lower earnings and educational attainment (4 out of 6 studies), as well as adverse cardiovascular (3 out of 6), respiratory (3 out of 3), psychiatric (7 out of 12), and anthropometric outcomes (2 out of 2), potentially leading to higher overall mortality (2 out of 3). The impact on female infants was more pronounced than on males in 8 out of 9 studies that differentiated by sex. The quality of evidence varied, being low for respiratory and longevity outcomes and very low for others.

The study by Weeda et al.^19^ presents a systematic review and meta-analysis examining the broad impact of climate change on child health. Temperature extremes (> 95th percentile of average daily temperature) and increased temperature variability are associated with increased emergency-department presentations, asthma incidence, and risk of infectious respiratory disease.

More research is needed to understand the long-term consequences of heat exposure during gestation, its biological pathways, especially in under-researched regions and as well as to understand how social inequalities exacerbate in-utero heat exposure effects on the health of the offspring.

In the case of air pollution exposure, there is more robust evidence regarding the consequences of exposure on postnatal health. A variety of negative health conditions are reported; the effects of air pollution extend beyond immediate birth outcomes. Gestational exposure to pollutants has been linked to congenital malformations and increased neonatal mortality rates.^20^ A systematic review^21^ and meta-analysis were conducted to assess recent evidence on the link between air pollution and congenital anomalies (CAs). Out of 11,014 records, 49 studies were included in the analysis. The results showed that for every 10 μg/m³ increase in air pollutants, exposure to PM10 during the first month of pregnancy and the first trimester was linked to a higher risk of overall congenital anomalies. Specifically, PM10 exposure during the first trimester was associated with congenital heart disease (odds ratio [OR] = 1.055) and patent ductus arteriosus (OR = 1.094). Additionally, exposure to PM10 throughout the entire pregnancy was linked to chromosomal anomalies, while exposure three months before pregnancy and during the first two months was associated with nervous system anomalies.

Recent studies also link prenatal exposure to Autism Spectrum Disorders,^22^ Attention deficit hyperactivity disorder (ADHD),^23^ increased risk of eczema^24^ or higher blood pressure.^25^

The study by Liu et al.^25^ is a systematic review and meta-analysis that investigates the effects of pre- and postnatal particulate matter (PM) exposure on blood pressure in children and adolescents. The analysis included a comprehensive evaluation of relevant studies to determine the relationship between PM exposure and blood pressure levels among youth. The results indicate that prenatal exposure to PM2.5 is linked to an increase in diastolic blood pressure (DBP) in offspring, with an average rise of 1.14 mmHg for every 10 μg/m³ increase in PM2.5 (95% CI: 0.12, 2.17). For short-term postnatal exposure, a 7-day average of PM2.5 was significantly associated with systolic blood pressure (SBP), showing an increase of 0.20 mmHg (95% CI: 0.16, 0.23), and DBP, which increased by 0.49 mmHg (95% CI: 0.45, 0.53). For long-term postnatal exposure, positive associations were found for SBP with PM2.5 (β = 0.44, 95% CI: 0.40, 0.48) and PM10 (β = 0.35, 95% CI: 0.19, 0.51). DBP also showed increases associated with PM1 (β = 0.45), PM2.5 (β = 0.31), and PM10 (β = 0.32). Furthermore, there was a significant association between particulate matter exposure and hypertension risk; for every unit increase in particulate matter, the odds of developing hypertension increased by factors of 1.43, 1.65, and 1.26 depending on the particulate matter fraction considered. The smaller the particle the greater the risk. The findings indicate that both prenatal and postnatal exposure to particulate matter is associated with elevated blood pressure in children and adolescents. Specifically, the results suggest that increased exposure to PM can lead to significant increases in systolic and diastolic blood pressure.

Although there is compelling evidence on the association between air pollution exposure and obesity among adults, gestational exposure is associated with obesity at 4 and 6 years only if mothers had inadequate fruit and vegetable intake.^26^ Siewert et al.^27^ found that in counties with low overall air pollution, there is low to no impact on exposure to childhood obesity, unlike countries with higher levels of pollution.

Neurodevelopment is also negatively influenced by prenatal and early life exposure to air pollution. Changes in structural morphology^28^ and impairments on intellective functioning, memory and learning, attention and executive functions, verbal language, numeric ability, and motor and/or sensorimotor functions are also affected.^29^

The study by Mishra et al.^30^ provides insights into how prenatal exposure to air pollution air pollution, can influence biological markers associated with aging. Their review focuses on how exposure to air pollutants, particularly particulate matter (PM2.5), affects telomere length (TL) and mitochondrial DNA (mtDNA) content in newborns, which are critical markers of biological aging. Are we aging before birth? Initial insights into the potential connection between prenatal air pollution, telomere length (TL), and mitochondrial DNA (mtDNA) content at birth were derived from the ENVIRONAGE (ENVIRonmental influence ON early AGEing) birth cohort. This research indicated that exposure to particulate matter (PM) during pregnancy was linked to shorter telomeres in both cord blood and placental samples.^31^

## Final considerations

It is essential to recognize the profound impact that environmental factors, such as air pollution and climate change, can have on maternal health, fetal development and neonatal health. The data presented underscores the significant risks that environmental pollution poses during gestation, influencing not only maternal health but also the short- and long-term well-being of the child.

Less evidence exists for climate-related factors,^32^ there are few studies, and findings are inconsistent. Another important gap is the scarcity of studies from LMICs. Most of the published studies investigated the impacts of climate in developed countries. Dalugoda et al.^32^ point out that if we consider that socioeconomic factors are potential modulators of these effects, we can expect greater health risks for low- and middle-income countries (LMICs).

From a pediatric perspective, this growing body of evidence highlights the need for a multifaceted approach to protecting maternal and child health. Equally concerning is the impact of climate change, particularly extreme heat and extreme weather events, which exacerbate risks during pregnancy. Climate related food insecurity and disruptions to healthcare access should not be forgotten, all pose significant threats to maternal and neonatal health, particularly in low- and middle-income countries where healthcare resources may already be stretched thin.

Pediatricians are in a unique position to advocate for policies that mitigate environmental risks and promote healthier living conditions for mothers and children. It is essential to educate parents, especially expectant mothers, about the dangers of air pollution and climate change, while encouraging preventive measures such as minimizing exposure to pollutants, ensuring proper nutrition, and promoting healthy coping strategies for stress.^33^

In addition to individual-level interventions, pediatricians should be active participants in advocating for broader public health initiatives that address the root causes of environmental pollution. This includes supporting regulations that limit emissions, ensuring access to clean air and safe environments, and collaborating with policymakers to create systems that support maternal and child health in the face of environmental challenges.

## Conclusion

The authors are observing a troubling beginning of life as a result of climate change and air pollution, with repercussions that can last a lifetime. It is imperative that we all take action to safeguard the health of future generations, ensuring a healthier environment for mothers and their children. Collective efforts are crucial in addressing these challenges and promoting the well-being of our communities.

## Financial support

Mariana M. Veras is supported by 10.13039/501100003593CNPq grant # 311576/2022–2.

## Conflicts of interest

The authors declare no conflict of interest.
